# Decoherence spectroscopy with individual two-level tunneling defects

**DOI:** 10.1038/srep23786

**Published:** 2016-03-31

**Authors:** Jürgen Lisenfeld, Alexander Bilmes, Shlomi Matityahu, Sebastian Zanker, Michael Marthaler, Moshe Schechter, Gerd Schön, Alexander Shnirman, Georg Weiss, Alexey V. Ustinov

**Affiliations:** 1 Physikalisches Institut, Karlsruhe Institute of Technology (KIT), 76131 Karlsruhe, Germany; 2 Department of Physics, Ben-Gurion University of the Negev, Beer Sheva 84105, Israel; 3 Institut für Theoretische Festkörperphysik, KIT, 76131 Karlsruhe, Germany; 4 Institut für Theorie der Kondensierten Materie, KIT, 76131 Karlsruhe, Germany; 5 L. D. Landau Institute for Theoretical Physics RAS, Kosygina street 2, 119334 Moscow, Russia; 6 National University of Science and Technology MISIS, Leninsky prosp. 4, Moscow, 119049, Russia; 7 Russian Quantum Center, 100 Novaya St., Skolkovo, 143025 Moscow region, Russia

## Abstract

Recent progress with microfabricated quantum devices has revealed that an ubiquitous source of noise originates in tunneling material defects that give rise to a sparse bath of parasitic two-level systems (TLSs). For superconducting qubits, TLSs residing on electrode surfaces and in tunnel junctions account for a major part of decoherence and thus pose a serious roadblock to the realization of solid-state quantum processors. Here, we utilize a superconducting qubit to explore the quantum state evolution of coherently operated TLSs in order to shed new light on their individual properties and environmental interactions. We identify a frequency-dependence of TLS energy relaxation rates that can be explained by a coupling to phononic modes rather than by anticipated mutual TLS interactions. Most investigated TLSs are found to be free of pure dephasing at their energy degeneracy points, around which their Ramsey and spin-echo dephasing rates scale linearly and quadratically with asymmetry energy, respectively. We provide an explanation based on the standard tunneling model, and identify interaction with incoherent low-frequency (thermal) TLSs as the major mechanism of the pure dephasing in coherent high-frequency TLS.

Although the existence of two-level tunneling systems in amorphous materials has been known for decades, they have attracted much renewed interest after their detrimental effect on the performance of microfabricated quantum devices was discovered. There is evidence that TLSs reside in surface oxides of thin-film circuit electrodes^1^, at disordered interfaces^2^, and in the tunnel barrier of Josephson junctions^3^. Since TLSs possess both electric and elastic dipole moments by which they couple to their environment, they generate noise in various devices ranging from microwave resonators and kinetic inductance photon detectors^4^ through single-electron transistors^5^ to even nanomechanical resonators^6^. In state-of-the-art superconducting qubits, interaction with individual TLSs constitutes a major decoherence mechanism, where they give rise to fluctuations in time^7^ and frequency^8^ of qubit relaxation rates. On the other hand, this strong interaction turns qubits into versatile tools for studying the distribution of TLS^9^,^10^, their physical origin^11^ and mutual interactions^12^ as well as their quantum dynamics^13^.

The omnipresence of TLSs interference is contrasted by a notable lack of certainty regarding the microscopic nature of the tunneling entity^14^. Figure 1a illustrates some proposed models of TLS formation in the amorphous tunnel barrier of a Josephson junction: the tunnelling of individual or small groups of atoms between two configurations^15^,^16^, displacements of dangling bonds, and hydrogen defects^17^. Near the interface with superconducting electrodes, TLSs may also arise from bound electron/hole Andreev states^18^ or Kondo-fluctuators^19^.

In this work, we present first direct measurements of the decoherence rates of individual TLSs in dependence of their strain-tuned internal asymmetry energy parameter. Our experiment provides unprecedented information about the spectrum of the environment to which a TLS couples and the nature of this coupling.

Without referring to a particular microscopic mechanism, the standard tunnelling model^20^,^21^ assumes the potential energy of TLSs to have the form of a double-well along a suitable configurational coordinate, giving rise to quantum mechanical eigenstates that are superpositions of the particle’s position as illustrated in Fig. 1b).

To study the quantum state evolution of individual TLSs, we exploit the strong interaction between a superconducting phase qubit and defects residing in the tunnel barrier of its Josephson junction^3^. Figure 1a shows the circuit schematic of the qubit, whose potential energy is tuned via an applied magnetic flux to adjust the energy splitting *E*_*q*_ between the two lowest qubit states as indicated in Fig. 1c. A TLS is read out by tuning the unexcited qubit into resonance, hereby realizing a coherent swap operation that maps the TLS’ quantum state onto the qubit^12^. Subsequently, a short flux pulse is applied to measure the qubit population probability *P*(|1〉)^22^ which directly reflects the population of the TLS’ excited state. In our case, the TLS signal is limited by energy relaxation that occurs in the qubit at a characteristic time of 
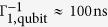
 during the readout sequence.

A probed TLS is characterized by the tunnelling energy Δ_*p*_ and the strain-dependent asymmetry energy *ε*_*p*_ (index *p* stands here for “probed”). In our experiments, we tune the asymmetry energy *in-situ* by slightly bending the sample chip using a piezo actuator^23^, resulting in *ε*_*p*_(*V*) = *η*_*p*_ · (*V* − *V*_0,*p*_) where *V* is the applied piezo voltage and *V*_0,*p*_ the voltage at which the probed TLS becomes symmetric. The coefficient *η*_*p*_ is given by *η*_*p*_ = *γ*_*p*_∂*ϵ*/∂*V*, where *γ*_*p*_ is the deformation potential which indicates how strongly the probed TLS couples to the applied strain. The strain is denoted by *ϵ* = *δL*/*L* and we estimate ∂*ϵ*/∂*V* ≈ 10^−6^/Volt based on results from a calibration of the piezo elongation per applied voltage and finite-elements-simulation of the mechanical chip deformation^23^. The TLS Hamiltonian reads


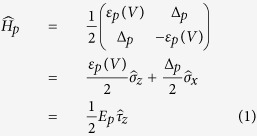


with the Pauli matrices 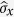 and 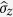. Diagonalization results in the energy difference between the TLS eigenstates 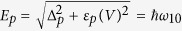, with Planck’s constant *ħ* and the TLS resonance frequency *ω*_10_. We defined the Pauli matrix 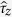 in the eigenbasis of the TLS, which acts on the eigenstates as 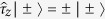.

## Experiment

**Figure 2:**
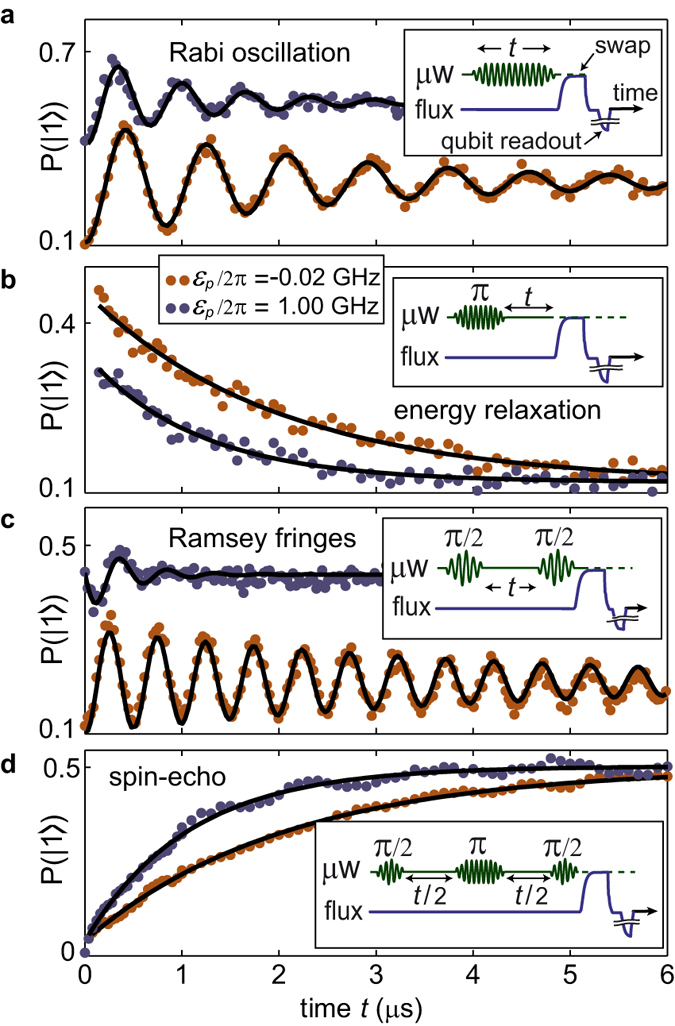
Quantum dynamics of TLS3. Each panel shows a measurement near the TLS symmetry point (red) and at *ε*_*p*_ = 2*π* × 1 GHz (blue). Insets depict the sequence of applied microwave (*μ*w) and flux pulses, where the latter realize a swap operation to map the TLS state onto the qubit plus a qubit readout pulse. (**a**) Rabi oscillations. (**b**) Energy relaxation to determine the *T*_1_ time. (**c**) Ramsey fringes to obtain the dephasing time *T*_2,*R*_. (**d**) Spin-echo measurement, resulting in the dephasing time *T*_2,*E*_. Blue curves in (**a**,**c**) were shifted by 0.3 for visibility. Panels (**a**–**c**) show raw data of the measured qubit population probability *P*(|1〉), whose reduced visibility is due to qubit energy relaxation during the TLS readout process.

**Figure 3:**
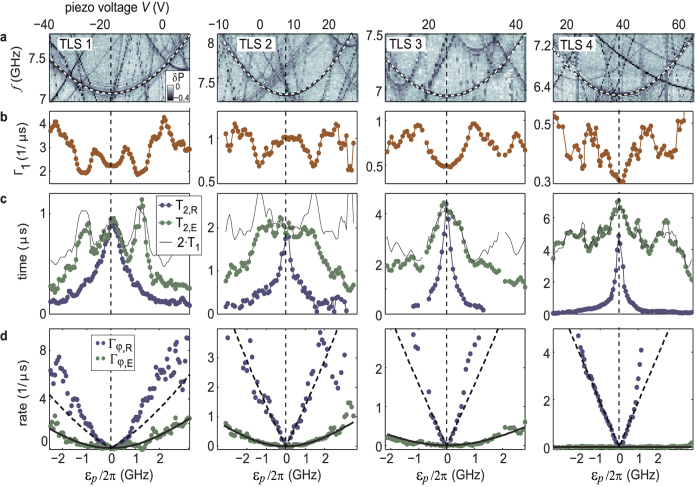
Spectroscopy and results of decoherence measurements, obtained on four TLSs. (**a**) Defect swap-spectroscopy, indicating the resonance frequencies of TLSs by a reduction *δP* of the qubit population probability (dark traces in color-coded data). Superimposed dots are obtained from microwave spectroscopy, to which hyperbolic fits (dashed lines) result in the static TLS parameters. (**b**) Energy relaxation rate Γ_1_. (**c**) Effective dephasing times *T*_2,*R*_ (blue) and *T*_2,*E*_ (green), measured using the Ramsey and spin-echo protocol, respectively. The thin black line indicates 2 · *T*_1_. (**d**) Pure dephasing rates calculated from the data in (**b**,**c**). Fitting curves (solid and dashed lines) are discussed in the text.

To measure TLS decoherence rates as a function of their asymmetry energy, we first apply our swap spectroscopy method^12^ to obtain an overview of the TLS frequency distribution in the sample. We then select a TLS whose symmetry point lies in the experimentally accessible strain range and perform microwave spectroscopy to calibrate its resonance frequency as a function of strain (see Fig. 3a). From a hyperbolic fit, we obtain the TLS’ tunnelling energy Δ_*p*_, its asymmetry *ε*_*p*_(*V*) as a function of the applied piezo voltage, and the deformation potential *γ*_*p*_. We then apply standard resonant microwave pulse sequences illustrated in the insets of Fig. 2 to observe the TLS’ coherent state evolution in the time domain^13^. After a calibration of the driving strength by observing Rabi oscillations (Fig. 2a), we measure the energy relaxation rate Γ_1_ ≡ 1/*T*_1_ by exciting the TLS with a *π*-pulse and fitting the decaying state population with an exponential ∝ exp(−Γ_1_ · *t*) as shown in Fig. 2b. The experimental results on Γ_1_ are summarized in Fig. 3b.

Next, we measure dephasing using the Ramsey (see Fig. 2c) and the spin-echo protocol (see Fig. 2d). In both of these protocols, the TLS is initalized into a superposition of the eigenstates using a *π*/2-pulse. The decay of this superposition assumes the general functional form exp[−Γ_1_*t*/2 − *x*_*i*_(*t*)], where *i* = *R* for Ramsey and *i* = *E* for spin-echo. The dephasing functions *x*_*R*_(*t*) and *x*_*E*_(*t*) depend strongly on the environment’s fluctuation spectrum and will be in the focus of our discussion below. Further details of these experiments are contained in Supplementary Information I.

Our time-domain data do not allow us to determine the exact functional form of the dephasing signal, because only a few oscillation periods are observed at asymmetry energies where Γ_*φ*,*R*_ dominates over Γ_1_ (see Fig. 2c). Since this renders fits to a linear dependence *x*_*i*_ = Γ_*φ*,*i*_*t* to appear practically indistinguishable from a Gaussian decay *x*_*i*_ = (Γ_*φ*,*i*_*t*)^2^, we estimate the pure dephasing rates Γ_*φ*,*i*_ in the linear approximation and deduce their functional form from their strain dependence. Figure set 3c summarizes the extracted effective dephasing times *T*_2,*R*_ = (Γ_1_/2 + Γ_*φ*,*R*_)^−1^ and *T*_2,*E*_ = (Γ_1_/2 + Γ_*φ*,*E*_)^−1^ of four different TLSs, which were measured in the same qubit sample. Using the previously obtained data on Γ_1_, we extract the pure dephasing rates Γ_*φ*,*R*_ and Γ_*φ*,*E*_, which are shown in Fig. 3d. Additional data obtained from other TLSs are included in the Supplementary Information I.

### Energy relaxation

For all investigated TLSs, we observe (see Fig. 3b) that their energy relaxation rates exhibit a strain-dependent structure that appears symmetric with respect to the point of lowest TLS energy *ε*_*p*_ = 0. This indicates that the spectral density of the underlying relaxing modes depends only on frequency and is independent of the applied strain. Therefore, we conclude that the dominant relaxation mechanism of the probed TLSs is not due to their near-resonant coupling to other TLSs, because those would also be detuned by the applied strain and thus are expected to generate non-symmetric patterns in Γ_1_. This notion is further supported by the finding that strong mutual TLS interactions are rarely observed for our sample^12^.

If the noise spectral density was constant around *ω* = Δ_*p*_/*ħ*, the strain dependence of Γ_1_ for 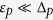 would be given (see Supplementary Information II) by 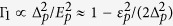, i.e. it would show a weak parabolic decrease around the symmetry point. As seen in Fig. 3b, such a scaling is obscured by the pronounced frequency-dependence of the noise spectral density. One may assume that this structure originates from the coupling to phonon modes which should have a discrete spectrum since the lateral size of the junction’s dielectric is comparable to the wavelength of high-frequency phonons. Indeed, a comparison of Γ_1_ of different TLS as a function of their resonance frequencies (see Supplementary Information I) reveals a common maximum at 7.4 GHz for 3 out of 5 investigated TLS, indicating that those TLS may be coupled to the same phononic mode^24^.

### Pure dephasing

The observed pure dephasing “rates” Γ_*φ*,*R*_ and Γ_*φ*,*E*_ show the following main features: a) the echo protocol is extraordinarily efficient, so that the ratios Γ_*φ*,*R*_/Γ_*φ*,*E*_ reach very large values (see Table 1). b) the *ε*_*p*_-dependence of the echo dephasing rate is clearly parabolic: 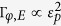; c) close to the symmetry point, the *ε*_*p*_-dependence of the Ramsey dephasing rate Γ_*φ*,*R*_ could be fitted to a linear behavior, Γ_*φ*,*R*_ ∝ |*ε*_*p*_|, in all TLSs. In the Supplementary Information II, we review shortly the well known results in order to identify possible sources of pure dephasing. We conclude that an environment characterized by white noise or by 1/*f* noise could not explain the experimental findings.

### Interpretation of the experimental results

We argue that the experimental observations can be explained in the framework of the standard tunnelling model^20^,^21^. In contrast to the probed high frequency TLS, whose energy splitting is much higher than the thermal energy, 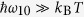, the TLSs responsible for pure dephasing are “thermal”, i.e. their energy splittings are lower than *k*_B_*T* so that they switch randomly between their states. We argue that the switching rates of thermal TLSs are very low, leading to the essentially non-Gaussian noise that has a spectral power more singular than 1/*f*. In this case, the Ramsey dephasing is dominated typically by the nearest neighbouring thermal TLS^25^,^26^,^27^. Since the asymmetry energies of the thermal TLSs also change with strain, one expects that some TLSs will go in and out of the group of relevant thermal TLSs as the strain is varied. Thus, the dominant decohering TLS will be replaced by another thermal TLS when the change of its asymmetry energy is on the order of the thermal energy *k*_*B*_*T* (that is, ~2*π* × 1 GHz). This gives rise to a non-regular behavior, reflected in a change of slope or small irregularities in the Ramsey dephasing rate as a function of strain as seen in Fig. 3d. Thus, we shall focus on a region of order |*ε*_*p*_| < *k*_*B*_*T* ≈ 2*π* × 1 GHz (here and in other places we use *ħ* = 1) near the symmetry point and study the dephasing by a single thermal TLS. In this scenario, close to the symmetry point Γ_*φ*,*R*_ ∝ |*ε*_*p*_| in the general case, or Γ_*φ*,*R*_ ∝ |*ε*_*p*_|^2^ in the special case where the decohering TLS is near its own symmetry point. The dominant thermal TLS is almost completely eliminated by the echo protocol. This explains the very high efficiency of the echo technique. We argue that the echo dephasing rate due to the thermal TLSs is much lower than the one due to the residual white noise environment, which explains the observed 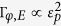.

### Theory

In the standard tunnelling model each isolated TLS is described by a Hamiltonian 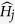 as in Eq. (1), with the index *p* replaced by *j*. The asymmetry *ε*_*j*_ and tunneling energy Δ_*j*_ are assumed to be randomly distributed with a universal distribution function 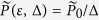, where 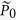 is a material dependent constant^15^. Each TLS is also characterised by its coupling *γ*_*j*_ to the strain field. Moreover, it is well established that TLSs interact via phonon-mediated interactions, which can be described by a low-energy effective Hamiltonian of the form^28^,^29^,^30^


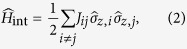


with the interaction coefficients


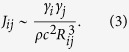


Here *R*_*ij*_ is the distance between the TLSs and *ρ, c* are the mass density and sound velocity, respectively. A central dimensionless parameter of the tunnelling model is the tunneling strength 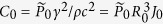, where *R*_0_ and 
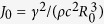
 are the typical distance and typical interaction strength between nearest neighbour TLSs, respectively. The well-known similarity in the low-temperature properties of disordered solids is reflected in a universal value of *C*_0_ ≈ 10^−3 ^31,32.

We consider now the probed TLS interacting with a set of thermal TLSs via the coupling mechanism of Eq. (2). Only the coupling terms involving the slow (non-rotating) variables of both the probed TLS, 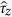, and of the thermal TLSs, 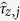, are relevant for pure dephasing. Moreover, at frequencies relevant for pure dephasing, the operators 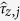 can safely be replaced by classical stochastic processes *τ*_*z*,*j*_(*t*) describing random switching between *τ*_*z*,*j*_ = ±1 with switching rate Γ_1,*j*_. Thus, the Hamiltonian of the probed TLS reduces effectively to


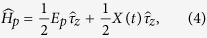


where 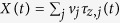. The effective couplings are given by


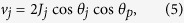


where 
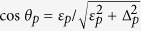
 and 
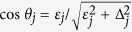
. Here *J*_*j*_ is the coupling strength (3) between the probed TLS and thermal TLS number *j*.

The theory of pure dephasing due to a coupling to an ensemble of TLSs is discussed in Supplementary Information II. Here we provide the qualitative estimates. The effect of a thermal TLS on the coherence properties of the probed TLS depends on the coupling *v*_*j*_ and on the switching rate (relaxation rate) Γ_1,*j*_ of the thermal TLS. We assume that the random transitions of each TLS are mainly due to their coupling to phonons, in which case the relaxation rate reads^21^,^33^


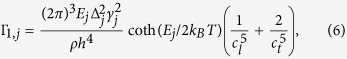


where *c*_*l*_ and *c*_*t*_ are the sound velocities of the longitudinal and transverse modes, respectively. The maximum switching rate 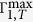 among the thermal TLS for which *E*_*j*_ ≤ *k*_B_*T* (the TLSs with *E*_*j*_ > *k*_B_*T* are not thermal and do not give rise to low frequency noise) is obtained for Δ_*j*_ = *E*_*j*_ and *E*_*j*_ = *k*_B_*T*. Setting *γ*_*j*_ ≈ 1 eV and *T* = 35 mK^10^,^15^,^23^, we obtain 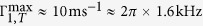.

Next, we estimate the typical coupling strength of the nearest thermal TLS, *J*_*T*_, by calculating the typical distance between the probed TLS and its nearest neighbouring thermal TLS in three and two dimensions (3D and 2D, respectively), and find (see Supplementary Information II)


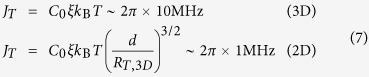


where 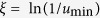, with *u*_min_ being a lower cutoff for the parameter *u* ≡ sin^2^ *θ* = (Δ/*E*)^2 ^34, *d* ≈ 3 nm is the thickness of the tunnel dielectric, and we assumed the usual values *C*_0_ ≈ 10^−3^ and *ξ* ≈ 20.

The above estimates reveal that thermal TLSs satisfy 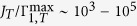. We recall the relation *v*_*j*_ = *J*_*j*_ cos *θ*_*j*_ cos *θ*_*p*_ and take into account that, typically, cos *θ*_*j*_ = *O*(1). Thus, the closest thermal TLS is in the strong coupling regime, 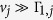, except in the very close vicinity of the symmetry point *ε*_*p*_ = 0 of the probed TLS. Therefore, we should study the *ε*_*p*_-dependence of the dephasing rates assuming the presence of strongly coupled thermal TLSs. Such a situation also provides an explanation for the effectiveness of the echo protocol. This is clearly illustrated by an example of dephasing caused by a single TLS with 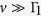 (we drop the TLS index *j*) discussed in ref. 26. While the Ramsey dephasing “rate” is of order *v*, the echo dephasing “rate” is of order Γ_1_ of the thermal TLS (see Supplementary Information II for details). Thus, in this case, Γ_*φ*,*R*_ ∝ |*ε*_*p*_|, whereas Γ_*φ*,*E*_ is independent of the applied strain.

The situation is more involved in the case of an ensemble of thermal TLSs. Since the coupling strength between the TLSs scales with their distance *r* as 1/*r*^3^, the closest thermal TLS dominates the Ramsey dephasing. Averaging the decay function over the distribution function of TLSs is not appropriate, i.e., there is no self-averaging (see Supplementary Information II). The typical Ramsey decay is approximately characterized by an envelope function 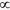 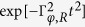, with possible few oscillations due to a small number of decohering TLSs^26^. The Ramsey dephasing “rate” reads


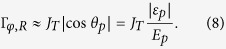


In deriving Eq. (8), we assumed that the factor cos *θ*_*j*_ in Eq. (5) for the closest thermal TLS does not depend strongly on the applied strain. This assumption is valid in the most probable case, where the closest thermal TLS is not expected to be close to its own symmetry point (*ε*_*j*_ = 0) at the piezo voltage *V* = *V*_0,*p*_ (i.e. at the same voltage for which the probed TLS is in its symmetry point). However, in the more special case in which the closest thermal TLS is near its symmetry point at *V* = *V*_0,*p*_, the Ramsey dephasing rate is expected to change quadratically with the piezo voltage, that is


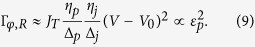


This special situation could be of relevance for TLS1. Indeed, as one can observe in the leftmost column of Fig. 3d, a parabolic fitting could be performed here in a wider range of *ε*_*p*_ as compared to the shown linear fit.

For the echo decay due to a single strongly coupled thermal TLS, the dephasing rate is independent of *ε*_*p*_. For an ensemble of TLSs it turns out that the decay function is not dominated by the closest TLS, but rather multiple TLSs contribute, i.e., there is self-averaging in this case (see Supplementary Information II). However, the theory predicts Γ_*φ*,*E*_ ∝ |*ε*_*p*_|^0.4^ and Γ_*φ*,*E*_ ∝ |*ε*_*p*_|^0.5^ in 2D and 3D, respectively, in disagreement with the experimental results that show a quadratic dependence of the echo dephasing rate. Moreover, the predicted order of magnitude is too small to explain the experimentally observed echo dephasing rate, i.e. the mechanism of interactions between TLSs is expected to yield echo efficiencies even stronger than those observed in our experiment. This is supported by the data obtained on TLS4 (see rightmost plot of Fig. 3d), for which the echo protocol is very efficient in the whole range of *ε*_*p*_.

To explain the experimental findings we are forced to assume some extra white noise environment that leads (see Supplementary Information II) to


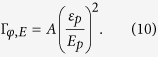


Such a white noise environment could result e.g. from fast relaxing TLSs^30^ or from non-equilibrium quasiparticles^35^,^36^. Quasiparticles are well known to induce decoherence in superconducting quantum devices. An estimate of the dephasing rate induced by non-equilibrium quasiparticles is in good agreement with the fitting parameter *A* (see Supplementary Information II).

The above contribution of the white noise [Eq. (10)] gives a similar contribution to the Ramsey dephasing rate. Thus, combining (8) with (10) we attempt to fit Γ_*φ*,*R*_ in Fig. 3d in the vicinity of the symmetry point (|*ε*_*p*_|/2*π* < 1 GHz) using


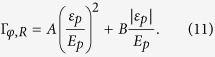


As explained above, one should not expect a pure linear or parabolic behavior of the Ramsey dephasing rate on the whole range of *ε*_*p*_. Being dominated by single thermal TLSs, the Ramsey dephasing rate is expected to exhibit a change of slope as the decohering TLSs go in and out of the set of thermal TLSs, that is when |*ε*_*p*_|/2*π* > 1 GHz. Yet, in a typical case, one expects a linear behavior in a narrow vicinity of the symmetry point.

Table 1 summarizes the fitting parameters *A* and *B* as well as other extracted TLS parameters. According to our theory, the parameter *B* is associated with the coupling *J*_*T*_ [Eqs (7) and (8)]. The estimations of the standard tunnelling model for *J*_*T*_ in the 2D case are in good agreement with the fitting parameter *B* for all TLSs.

Summarizing, our measurements of TLS decoherence rates as a function of their asymmetry energy reveal that TLS relaxation occurs mainly due to their coupling to discrete phonon modes, while dephasing is dominated by their interaction with randomly fluctuating thermal TLS at low energies. Our theory predicts that thermal TLSs in the standard tunnelling model are characterized by 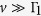, i.e. their coupling strength to the probed TLS exceeds their switching rate. Such TLSs produce noise which gives rise to an approximately linear dependence of the Ramsey dephasing rate of coherent TLS on the external strain (which, in more special cases, can also be quadratic). The Ramsey dephasing is dominated by a small number of thermal TLSs, which explains the observed irregularities in the Ramsey dephasing rate as a function of external strain. The order of magnitude of the measured Ramsey dephasing rate is in agreement with the theory. The strain dependence of the echo dephasing rate, on the other hand, can not be accounted for by the standard tunnelling model. Its explanation requires the presence of a white noise environment. This could consist e.g. of much faster fluctuators that are characterized by a weak interaction with the probed TLS, or non-equilibrium quasiparticles in the superconducting layers.

## Methods

The phase qubit sample used in this work was fabricated in the group of J. M. Martinis at University of California, Santa Barbara (UCSB), as described in ref. 37. The qubit junction had an area of about 1 *μ*m^2^, fabricated using aluminum as electrode material and its thermally grown oxide as a tunnel barrier. All data have been obtained at a sample temperature of about 35 mK. The mechanical strain was controlled by bending the sample chip with a piezo transducer as explained in ref. 23.

## Additional Information

**How to cite this article**: Lisenfeld, J. *et al*. Decoherence spectroscopy with individual two-level tunneling defects. *Sci. Rep.* **6**, 23786; doi: 10.1038/srep23786 (2016).

## Supplementary Material

Supplementary Information

## Figures and Tables

**Figure 1:**
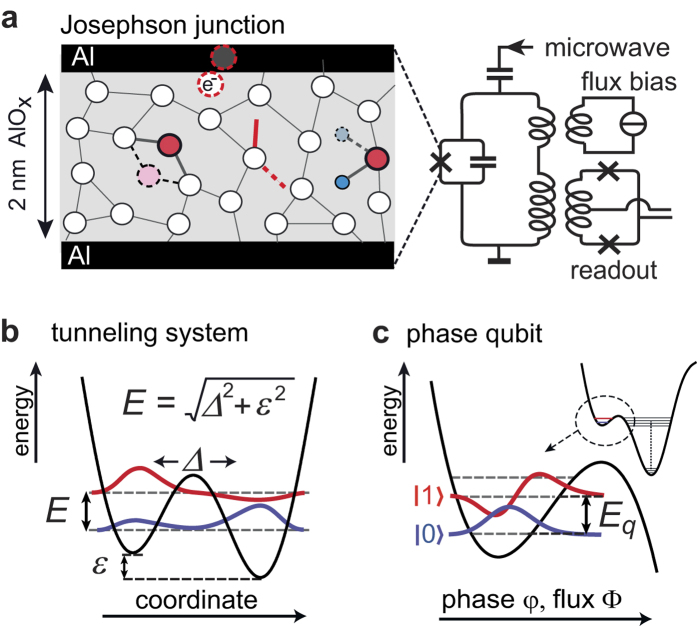
Models of two-level systems (TLSs) in the Josephson junction of a superconducting qubit. (**a**) Schematic of the phase qubit circuit used in this work and illustration of proposed TLS mechanisms: tunnelling atoms, trapped electrons, dangling bonds, and hydroxide defects. (**b**) Sketch of the TLS eigenfunctions in a double-well potential that is characterized by the strain-dependent asymmetry energy *ε* and the tunnel coupling Δ. (**c**) Potential energy and indication of the two lowest eigenstates of the phase qubit.

**Table 1:** Measured TLS parameters.

TLS	Δ_*p*_/2*π* (GHz)	(∂*ε*_*p*_/∂*V*)/2*π* (MHz/V)	*V*_0,*p*_ (V)	*D*_||_ (eÅ)	*T*_1_ @ *ε*_*p*_ = 0 (*μs*)	*A* (*μs*)^−1^	*B* (*μs*)^−1^	Γ_*φ*,*R*_/Γ_*φ*,*E*_
1	7.075	115.5	−18.01	0.37	0.44	14	7.7	8
2	7.335	180.3	7.64	0.29	0.99	4.4	9.1	17
3	6.947	156.7	24.10	0.26	2	3.3	10.5	22
4	6.217	146.8	38.65	0.46	3.2	0.0	13.3	∞

Static values Δ_*p*_, ∂*ε*_*p*_/∂*V* and *V*_0,*p*_ are obtained from a spectroscopic fit of *ω*_10_(*V*). *D*_‖_ is the component of the TLS’ dipole moment parallel to the electric field in the junction, extracted from the measured coupling strength to the qubit. *T*_1_ is quoted at the TLS’ symmetry point. Parameters *A* and *B* result from fits of the measured dephasing rates in the region |*ε*_*p*_|/2*π* < 1 GHz to the spin-echo dephasing rate Γ_*φ*,*E*_ = *A* · (*ε*_*p*_/*E*_*p*_)^2^ and Ramsey dephasing rate Γ_*φ*,*R*_ = *A* · (*ε*_*p*_/*E*_*p*_)^2^ + *B* · (|*ε*_*p*_|/*E*_*p*_), respectively. The last column gives the approximate ratio between Ramsey and echo rates, estimated in the region |*ε*_*p*_|/2*π* < 1 GHz.
