# Supplementary material for: Decoherence spectroscopy with individual two-level tunneling defects
Source: Sci Rep. 2016 Mar 31;6:23786. doi: 10.1038/srep23786 (PMC4815015; doi:10.1038/srep23786)
Supplement: Supplementary Information [file srep23786-s1.pdf]

Decoherence spectroscopy with individual two-level tunneling defects
Supplementary Material

Jürgen Lisenfeld,[1] Alexander Bilmes,[1] Shlomi Matityahu,[2] Sebastian Zanker,[3] Michael Marthaler,[3] Moshe Schechter,[2] Gerd Schön,[3] Alexander Shnirman,[4,5] Georg Weiss,[1] and Alexey V. Ustinov[1,6]

[1]*Physikalisches Institut, Karlsruhe Institute of Technology (KIT), 76131 Karlsruhe, Germany*
[2]*Department of Physics, Ben-Gurion University of the Negev, Beer Sheva 84105, Israel*
[3]*Institut für Theoretische Festkörperphysik, KIT, 76131 Karlsruhe, Germany*
[4]*Institut für Theorie der Kondensierten Materie, KIT, 76131 Karlsruhe, Germany*
[5]*L. D. Landau Institute for Theoretical Physics RAS, Kosygina street 2, 119334 Moscow, Russia*
[6]*National University of Science and Technology MISIS, Leninsky prosp. 4, Moscow, 119049, Russia*

I. EXPERIMENT

A. Qubit sample

In this work, we investigate TLS which are residing in the amorphous tunnel barrier of the Josephson junction in a superconducting phase qubit. The qubit sample was fabricated by the group of J.M. Martinis in the year 2005 and is described in Ref. [1]. The tunnel barrier of the qubit junction consists of an approximately 3 nm-thick thermally oxidized amorphous aluminum oxide layer, with a junction area of about 1 μm^2. An additional on-chip shunting capacitor that contains low-loss SiNx as dielectric provides most of the qubit's capacitance of 850 fF, and a gradiometric coil of approximate footprint 100x100 μm^2 contributes the inductance (cf. the circuit schematic in Fig. 1 of the main text). Since the tunnel barrier of the junction is about two orders of magnitude thinner than the dielectric of the shunt capacitor, we expect that only TLS residing in the junction experience electric fields sufficiently large to become strongly coupled to the qubit.

All presented data have been acquired at a sample temperature of 35 mK. The qubit had an energy relaxation time of $T_1 \approx 100$ ns and similar effective dephasing time T_2.

B. TLS spectroscopy

We detect the resonance frequencies of TLS by the swap-spectroscopy method discussed in Ref. [2]. For this, we apply the pulse sequence depicted in the inset to Fig. **S1**: the qubit is first excited by a resonant π-pulse and subsequently tuned to variable probing frequencies via an applied flux pulse of fixed duration, and then read out via a short flux pulse. During the detuning pulse, the qubit may interact with TLS that have their resonance at the chosen probing frequency. In the case of being resonant with a strongly coupled TLS, oscillations will occur in the qubit's excitation probability $P(|1\rangle)$ which reflect the coherent energy redistribution in the coupled qubit-TLS system. For weakly coupled TLS, or in the case when the TLS coherence time is much shorter than that of the qubit, instead of oscillations a reduction in $P(|1\rangle)$ is expected after the two systems have interacted. By plotting the changes in qubit population probability δP vs. both frequency (i.e. amplitude of the applied detuning pulse) and mechanical strain (i.e. applied piezo voltage), we obtain a detailed overview of TLS resonance frequencies as shown in Fig. **S1**. The traces of the TLS whose coherent dynamics were studied for this work are highlighted by colour in Fig. **S1**.

C. Observing coherent TLS dynamics

We manipulate the TLS quantum state using large amplitude microwaves tuned to the TLS resonance frequency and applied via an on-chip microwave line that is coupled capacitively to the hosting Josephson junction. Hereby, the TLS absorbs photons via a Raman type transition that involves a virtual excitation of higher excited phase qubit states [3]. While the TLS is driven, we keep the qubit detuned by more than 1 GHz from the TLS resonance to ensure that residual coupling does not reduce TLS coherence.

When qubit and TLS are tuned in resonance, energy that was initially stored in just one subsystem will redistribute, giving rise to coherent oscillations of the excitation between TLS and qubit. To measure the TLS state, the qubit (which was initially prepared in its ground state) is pulsed into the TLS resonance for a time that equals half the inverse qubit-TLS coupling strength, hereby realizing an iSWAP operation that swaps the states of qubit and TLS.

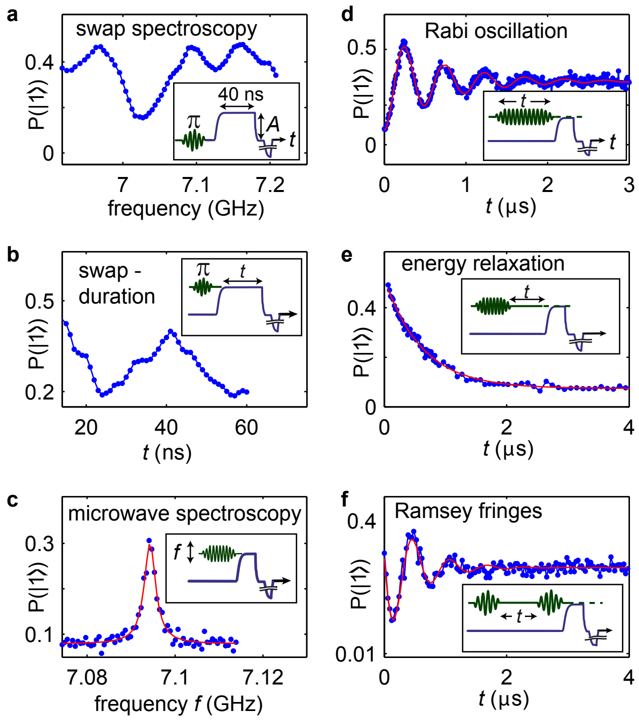

Figure S1. **Sequence of measurements to characterize TLS coherence at a given value of applied strain.** **a** Swap spectroscopy to find the optimal swap pulse amplitude for TLS readout, here given by the minimum in $P(|1\rangle)$. **b** Variation of the interaction time between qubit and TLS to find the optimal swap duration, which is given by the first minimum in $P(|1\rangle)$. **c** TLS spectroscopy, obtained by applying a long microwave pulse of varying frequency and measuring the resulting TLS population probability. **d** Rabi oscillation measurement. **e** Measurement of the energy relaxation time T_1. **f** Measurement of Ramsey fringes.

Afterwards, the qubit is read out via a nanosecond-long flux pulse that maps its phase eigenstates states to different numbers of flux quanta in the qubit inductance, which are finally distinguished by performing a switching-current measurement of the DC-SQUID [4].

To measure TLS coherence times, at each strain an automated script first roughly detects the TLS resonance by the defect spectroscopy protocol as shown in Fig. **S1**a. For this, the qubit is excited and tuned by a flux pulse of varying amplitude near the expected TLS resonance frequency, where it is kept for a duration of about the swap duration. A minimum in the resulting qubit population then indicates the optimal detuning pulse amplitude at which most energy has been transferred between the systems. Figure **S2** shows an overview of defect spectroscopy in a wider range of frequency and mechanical strain, where the traces of the TLS whose decoherence properties are studied in this work are highlighted by colours.

Optionally, in the next step we re-calibrate the optimal swap duration by varying the time for which the qubit is tuned into resonance with the TLS. The swap duration at which the qubit excitation is maximally transferred is taken as the first minimum in the data as shown in Fig. **S1**b.

Next, we record the resonance curve of the TLS (Fig. **S1**c), for which we sweep the frequency of a long microwave pulse around the TLS resonance as it was roughly determined by previous swap spectroscopy. Fitting to a Lorentzian results in the exact TLS resonance frequency which is used for subsequent measurements.

From a Rabi protocol (Fig. **S1**d), the duration of π and $\pi/2$ pulses are calibrated for subsequent measurements of TLS decoherence times. The energy relaxation time T_1 is obtained from an exponential fit to the decaying TLS population after it was excited by a π-pulse as shown in Fig. **S1**e.

We measure TLS dephasing using a Ramsey sequence in which the delay between two $\pi/2$-pulses is varied. Here, the microwave frequency is detuned by 3 MHz from the exact TLS resonance to obtain an oscillatory signal of the TLS population as a function of pulse delay. The effective dephasing time $T_{2,R}$ is estimated by fitting the oscillation envelope to an exponential $\propto \exp(-t/T_2)$, for more details see the main text.

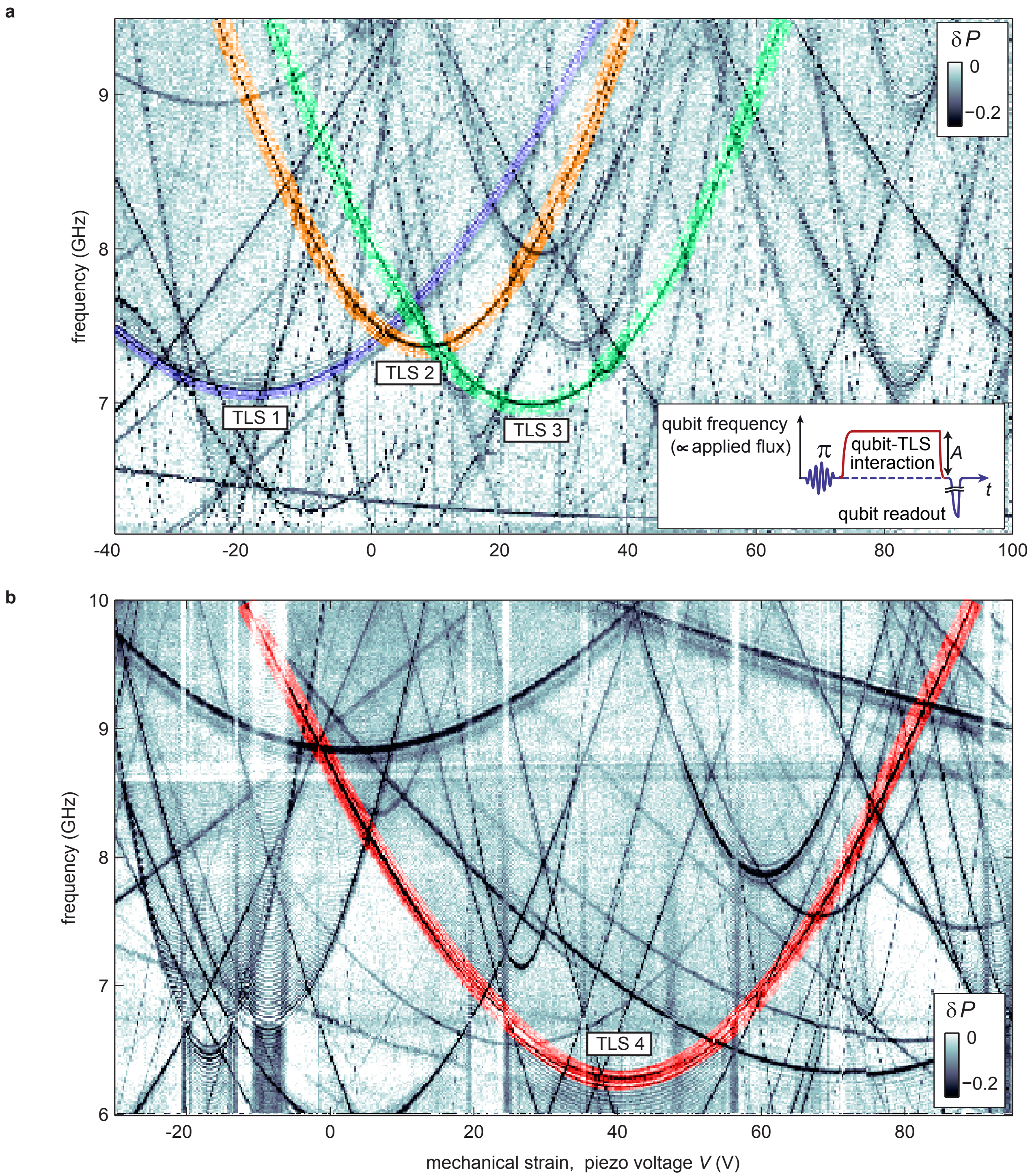

Figure S2. **Defect spectroscopy.** The strain-tuned TLS resonance frequencies are observed as dark traces which indicate a reduction in qubit population δP due to resonant TLS interaction. **a** The resonance frequencies of TLS 1, TLS 2 and TLS 3 are highlighted by colours. The inset depicts the sequence of qubit operations: excitation by a microwave π-pulse, tuning to a probe frequency using a flux pulse of variable amplitude but fixed duration, and a qubit readout pulse. **b** Data acquired from the same sample and experimental setup as in **a**, but in a different cool-down and using higher resolution. The red highlighted hyperbola indicates the trace of TLS 4.

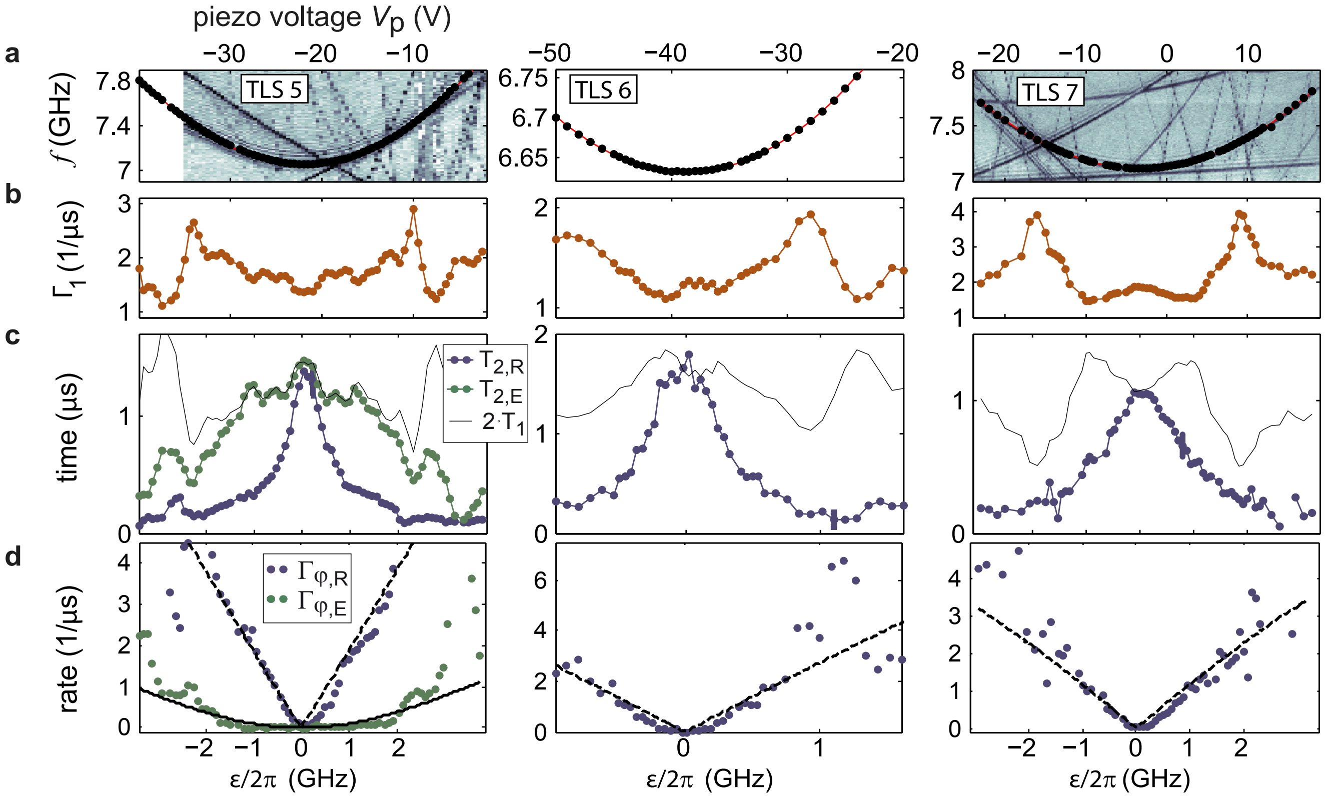

Figure S3. Spectroscopy and results of decoherence measurements, obtained on three additional TLS. **a** Swap spectroscopy (grayscale data) and TLS spectroscopy (black dots), with hyperbolic fits (red lines). **b** Energy relaxation rate Γ_1. **c** Effective dephasing time T_2 obtained in Ramsey (blue points) and spin-echo measurements (green points). **d** Pure dephasing rates obtained from Ramsey (blue) and spin-echo (green) sequences, with fits as described in the main text.

D. Spin-echo measurements

For spin-echo measurements, we employ the so-called phase-cycling technique [5] to avoid oscillations on the dephasing signal that originate in imperfectly calibrated $\pi/2$ and π-pulses. The spin-echo signal is recorded for several combinations of Bloch-vector rotations about different axes. From a total set of 8 spin-echo curves, those are combined in which effects of pulse imperfections appear with equal amplitude but opposite sign in order to reconstruct the unperturbed phase decay. This technique works very well also for scripted automatic measurements without adding a large work overhead to calibrate the IQ-mixer used for microwave pulse synthesis.

In our experiments, because of the small effective TLS-microwave coupling, we have to use relatively long refocussing π pulses (100 - 200 ns), and this gives rise to a residual small sensitivity of our spin-echo measurements to noise at low frequencies.

E. Decoherence properties of additional TLS measured in this work

We include data obtained on three further TLS in Figure **S3**. These additional TLS show the same features as those discussed in the main text: the strain dependence of the energy relaxation rate displays a frequency-dependent structure that is symmetric with respect to the TLS' degeneracy point $\varepsilon_p = 0$; the pure dephasing rate $\Gamma_{\varphi,\mathrm{R}}$ vanishes at the TLS' symmetry point and scales linearly with ε_p in its vicinity. For TLS 6 and TLS 7, we did not measure their dephasing rates under the spin-echo protocol. The extracted parameters of those additional measurements are contained in Table **S1**.

TLS	$\Delta_p/2\pi$ (GHz)	$(\partial\varepsilon_p/\partial V)/2\pi$ (MHz/V)	$V_{0,p}$ (V)	$D_\parallel$ (eÅ)	T_1 @ $\varepsilon_p = 0$ (μs)	A $(\mu s)^{-1}$	B $(\mu s)^{-1}$	Γ_R/Γ_E
5	7.056	190.5	-22.18	0.50	0.73	5.0	12.6	18
6	6.632	86.0	-38.76	0.38	0.82	–	18.2	–
7	7.118	150.3	-3.27	0.28	0.53	–	8.4	–

Table S1. Extracted parameters from the measurements in Fig. **S3**. Static values $\Delta_p, \partial\varepsilon_p/\partial V$ and $V_{0,p}$ are obtained from a spectroscopic fit of $\omega_{10}(V)$. $D_\parallel$ is the component of the TLS' dipole moment parallel to the electric field in the junction, extracted from the measured coupling strength to the qubit. T_1 is quoted at the TLS' symmetry point. Parameters A and B result from fits of the measured dephasing rates in the region $|\varepsilon_p|/2\pi < 1$ GHz to the spin-echo dephasing rate $\Gamma_{\varphi,E} = A \cdot (\varepsilon_p/E_p)^2$ and Ramsey dephasing rate $\Gamma_{\varphi,R} = A \cdot (\varepsilon_p/E_p)^2 + B \cdot (|\varepsilon_p|/E_p)$, respectively. The last column gives the approximate ratio between Ramsey and echo rates, estimated in the region $|\varepsilon_p|/2\pi < 1$ GHz.

F. Frequency dependence of TLS energy relaxation

For all seven investigated TLS, we observe that their energy relaxation rate exhibits a strain-dependent structure that appears symmetric with respect to the TLS' symmetry point $\varepsilon_p = 0$ (see Fig. 1a). This indicates that the dominant relaxation mechanism of the observed TLS is not due to their coupling to other TLS, because those would also be detuned by the applied strain and thus generate a non-symmetric pattern in Γ_1. In contrast, the frequency-dependence of Γ_1 may originate in the coupling to phonon modes, which have a discrete spectrum since the geometrical size of the junction dielectric of $\approx 1\mu$m is comparable to the wavelength of high-frequency phonons.
By plotting the TLS energy relaxation rates as a function of frequency, we test whether the TLS' noise spectral densities $S(\omega)$ extracted from their energy relaxation rates $\Gamma_1(\omega)$ show common features, i.e. whether peaks or dips in $S(\omega)$ appear at similar frequencies for different TLS. This could be seen as indication that different TLS experience the same phononic spectrum. For the energy relaxation rate,

$$\Gamma_1 \equiv 1/T_1 = \pi S_\omega(\omega) = \frac{\pi}{\hbar^2}\left(\frac{\partial\varepsilon(V)}{\partial V}\sin\theta\right)^2 S_V(\omega), \tag{S1}$$

where $S_V(\omega)$ is the symmetrized voltage noise spectral density at the TLS transition frequency $\omega = E_p/h$ that is converted to $S_\omega(\omega)$ by multiplication with the spectroscopically determined deformation potential $\partial\varepsilon_p/\partial V$, and $\sin\theta = \Delta_p/E_p$ is the transverse matrix element. In Fig. **S4**, we plot $\Gamma_1/\sin^2\theta$. A maximum in energy relaxation rate is found at 7.4 GHz for TLS 1, 5 and 7, while for other TLSs no data is available in this frequency range or they don't show a pronounced peak here.

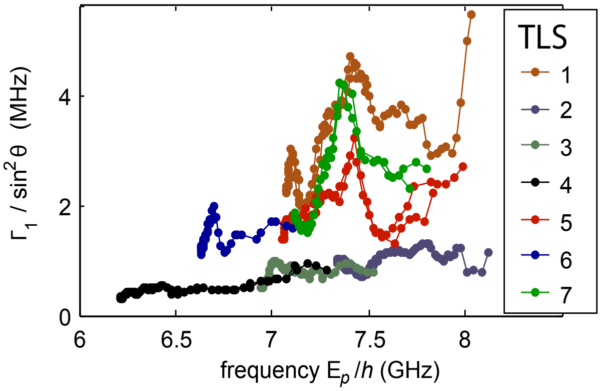

Figure S4. **TLS energy relaxation rate vs. frequency.** The plot of $\Gamma_1/\sin^2\theta$ as a function of frequency indicates a common maximum in energy relaxation rates of TLS 1, 5, and 7.

II. THEORY

A. Relaxation by phonons.

The most straightforward assumption about the TLS-phonon coupling would be of the form $H_{ph} = \gamma_p \hat{\sigma}_z \hat{\phi}$, where $\hat{\phi}$ is the phonon field related to strain. This would lead to the relaxation rate $\Gamma_1 = 2\gamma_p^2 (\Delta_p/E_p)^2\, S_\phi(\omega_{10})$, where $S_\phi(\omega) \equiv (1/2)\int dt\, e^{i\omega t}\, \langle \hat{\phi}(t)\hat{\phi}(0) + \hat{\phi}(0)\hat{\phi}(t)\rangle$ is the symmetrized noise spectral density of phonons. (In general $\Gamma_1 \propto |\mathbf{M}_1|^2 S(\omega_{10})$, where $\mathbf{M}_1 = \langle +|\hat{O}_1|-\rangle$ is the off-diagonal matrix element of the TLS operator $\hat{O}_1$ to which the environment couples and $S(\omega)$ is the spectral density of environment fluctuations. For strain related coupling to phonons, $\hat{O}_1 = \hat{\sigma}_z$ and $\mathbf{M}_1 = \Delta_p/E_p$.)

B. Standard theories of pure dephasing.

We start by summarizing the results for Gaussian noise. For the coupling Hamiltonian $H_\varphi = \hat{O}_\varphi \hat{X}$, where $\hat{O}_\varphi$ is a TLS observable (most generally a combination of Pauli matrices) and $\hat{X}$ is the observable of the low frequency environment having Gaussian statistics, the Ramsey and echo dephasing functions are given by

$$x_R(t) = \frac{\mathbf{M}_\varphi^2 t^2}{2} \int \frac{d\omega}{2\pi}\, S_X(\omega)\, \mathrm{sinc}^2\left(\frac{\omega t}{2}\right) , \tag{S2}$$

$$x_E(t) = \frac{\mathbf{M}_\varphi^2 t^2}{2} \int \frac{d\omega}{2\pi}\, S_X(\omega)\, \sin^2\left(\frac{\omega t}{4}\right) \mathrm{sinc}^2\left(\frac{\omega t}{4}\right) , \tag{S3}$$

where $S_X(\omega) \equiv (1/2)\int dt\, e^{i\omega t}\, \langle \hat{X}(t)\hat{X}(0) + \hat{X}(0)\hat{X}(t)\rangle$, and the relevant matrix element is given by $\mathbf{M}_\varphi = \langle +|\hat{O}_\varphi|+\rangle - \langle -|\hat{O}_\varphi|-\rangle$. Depending on the form of $S_X(\omega)$, the exact time dependence of $x_i(t)$ can vary [11]. An exponential decay, $x_i = \Gamma_{\varphi,i} t$, is obtained if $S_X(\omega)$ is approximately constant for $|\omega| \lesssim \Gamma_{\varphi,i}$ (this is a self-consistent condition), i.e., for white noise. Then, however, $\Gamma_{\varphi,E} \approx \Gamma_{\varphi,R} \approx (1/2)\mathbf{M}_\varphi^2 S_X(\omega = 0)$, i.e. the echo technique is inefficient. On the other hand, the quadratic dependence of $\Gamma_{\varphi,E}$ on ε_p can be explained if we assume that the environment couples to the dipole element of the TLS, i.e., $\hat{O}_\varphi = \hat{\sigma}_z/2$. This yields $M_\varphi^2 = \varepsilon_p^2/E_p^2$.

For frequency-dependent $S_X(\omega)$, which does not diverge at $\omega \to 0$, one might argue, based on Eqs. (S2) and (S3), that the dephasing rates are given by

$$\Gamma_{\varphi,R} = \frac{1}{2}\,\mathbf{M}_\varphi^2 S_X(\omega \approx 0) , \tag{S4}$$

$$\Gamma_{\varphi,E} = \frac{1}{2}\,\mathbf{M}_\varphi^2 S_X(\omega \approx 2\pi/T_E) , \tag{S5}$$

where T_E is the time delay between the echo pulses. This distinction is, however, only meaningful if $T_E \ll \Gamma_{\varphi,i}^{-1}$, i.e., if the time interval between the echo pulses is much shorter than the typical dephasing time. This is the case, e.g., in the "bang-bang" protocol [12]. In our experiment, however, $T_E \sim \Gamma_{\varphi,i}^{-1}$. Thus, the spectral densities in both Eqs. (S4) and (S5) should be understood as averaged over a frequency domain of order $\Gamma_{\varphi,i}$ and we obtain again $\Gamma_{\varphi,E} \approx \Gamma_{\varphi,R}$.

For $1/f$ Gaussian noise, $S_X(\omega) = A/f = 2\pi A/\omega$, the results are also well known [11]. In this case $x_R(t) \approx \mathbf{M}_\varphi^2 t^2 A \ln(1/(\omega_{ir} t))$ and $x_E(t) \approx \mathbf{M}_\varphi^2 t^2 A \ln 2$. Here ω_{ir} is the infra-red cut-off, which is usually identified with the time of averaging, $\omega_{ir} \sim 1/T_{av}$, that is with the time it takes to produce a single experimental point by averaging the results of multiple experimental runs. The dephasing "rates" can be defined via $x_i(t) \sim \Gamma_{\varphi,i}^2 t^2$. This gives with logarithmic accuracy $\Gamma_{\varphi,R}/\Gamma_{\varphi,E} \sim \sqrt{\ln(T_{av}/\tau_{\varphi,R})}$. With $T_{av} \sim 1$s and $\tau_{\varphi,R} \sim 1\mu$s we should expect $\Gamma_{\varphi,R}/\Gamma_{\varphi,E} \lesssim 4$. Thus, $1/f$ noise cannot explain the very high efficiency of the echo protocol observed in the experiment. In addition, we have $\Gamma_{\varphi,i} \propto |\mathbf{M}_\varphi| \propto |\varepsilon_p|$, which contradicts the experimental findings for the echo decay.

C. Theory of pure dephasing based on the standard tunneling model.

For the system described by the Hamiltonian (4) the decays of the Ramsey and echo signals are governed by $F_{R/E}(t) = \langle e^{i\varphi_{R/E}(t)}\rangle$, where $\varphi_R(t) = -\int_0^t X(t')dt'$ and $\varphi_E(t) = -\int_0^{t/2} X(t')dt' + \int_{t/2}^t X(t')dt'$ are the random phases accumulated at time t, respectively. The averaging is over the switching trajectories $X(t)$ of the set of thermal TLSs in a given disorder configuration.

For a single fluctuator, characterized by the switching rate Γ and coupling v, an average over the switching trajectory yields [6–8]

$$\begin{aligned} f_R(t) &= e^{-\Gamma t}\left(\cos\mu t + \frac{\Gamma}{\mu}\sin\mu t\right) \ , \\ f_E(t) &= e^{-\Gamma t}\left[1 + \frac{\Gamma}{\mu}\sin\mu t + \frac{\Gamma^2}{\mu^2}\left(1-\cos\mu t\right)\right] \ , \end{aligned} \qquad \text{(S6)}$$

where $\mu = \sqrt{v^2 - \Gamma^2}$. The decay function due to a set of fluctuators is the product of individual contributions of the form (S6), that is $F_{R/E}(t) = \prod_j f_{R/E,j}(t)$. It depends on the disorder configuration.

We estimate, first, the average over the disorder configurations of the TLSs, $\langle \ln|F_{R/E}(t)|\rangle_D$. Since we are dealing with quenched disorder, the question about the validity of self-averaging arises [9]. One should, therefore, examine whether the result for $\langle \ln|F_{R/E}(t)|\rangle_D$ corresponds to a typical situation. We obtain

$$\begin{aligned} &\langle \ln|F_{R/E}(t)|\rangle_D \\ &= \int d\Omega \int_{R_0}^{\infty} r^{D-1}dr \int_{u_{\min}}^{1} du \int_0^T dE\, P(E,u)\, \ln|f_{R/E}| \ , \end{aligned} \qquad \text{(S7)}$$

where D is the spatial dimension of the amorphous tunnel barrier (either bulk, $D = 3$, or thin layer, $D = 2$, of thickness d). Following the usual convention [10], we introduced

$$P(E,u) = \frac{P_0}{2u\sqrt{1-u}} \ , \qquad \text{(S8)}$$

which is derived from the standard tunneling model distribution function $P(\varepsilon,\Delta) = P_0/\Delta$ via the relations $E \equiv \sqrt{\varepsilon^2 + \Delta^2}$ and $u \equiv \sin^2\theta = (\Delta/E)^2$ with lower cutoff $u_{\min}$.

The functions $f_{R/E}(v,\Gamma)$ depend on Γ, given by Eq. (6), and the coupling constant $v(u,r)$. The latter depends on the parameter u and on the disorder configuration which determines the distance r between the probed TLS and the thermal ones via Eq. (3).

We now show that for the Ramsey protocol in the considered limit $v \gg \Gamma$ self-averaging, as implied by Eq. (S7), is not appropriate. (A similar conclusion had been reached in Ref. [9], although the present situation is more complicated by the fact that Γ and v are not independent.) To this end it suffices to consider the regime $t \ll 1/\Gamma_{1,T}^{\max}$, which is also the experimentally relevant time scale. In this limit dephasing is due to the averaging over the uncertainty of the initial state of the thermal TLSs.

In the considered limit we can replace f_R in Eq. (S7) by $\cos(vt)$. Due to the strong dependence, $v \propto 1/r^3$, and the fact that r can be much smaller than the typical distance, R_T, between the probed TLS and its nearest thermal TLS (estimated below), the averaging is strongly influenced by the tail of the distribution where $v \gg J_T$. Performing the integration we obtain

$$\begin{aligned} \langle \ln|F_R(t)|\rangle_D &\approx -J_T|\cos\theta_p|\,t & \text{for } D = 3 \ , \\ \langle \ln|F_R(t)|\rangle_D &\approx -\left[J_T|\cos\theta_p|\right]^{2/3} t^{2/3} & \text{for } D = 2 \ , \end{aligned} \qquad \text{(S9)}$$

where J_T is defined in (7) for both $D = 3$ and $D = 2$.

On the other hand, in a typical situation there is a closest thermal TLS with maximum coupling $v_T^{\max} \sim J_T|\cos\theta_p| \gg \Gamma_{1,T}^{\max}$. At short times, $t \ll 1/v_T^{\max}$, one obtains $\ln|F_R(t)| = -(1/2)t^2\sum_j v_j^2$. Since $v_j \sim 1/r_j^3$, with r_j being the distance between the probed TLS and the thermal TLS j, the sum is dominated by the few closest thermal TLSs and in both $D = 3$ and $D = 2$, and we obtain the typical result

$$\ln|F_R^{typ}(t)| \approx -[J_T\cos\theta_p]^2\, t^2 \ . \qquad \text{(S10)}$$

Both the average (S9) and the typical (S10) results give the dephasing "rate" (8), but with a very different functional time-dependence. At short times, $t \ll 1/\Gamma_{\varphi,R}$, the typical result gives a much weaker Ramsey decay than would be naively expected from the average result. At longer times, $t \gg 1/\Gamma_{\varphi,R}$, the difference between the average and the typical results is even more striking. The typical decay function $F_R^{typ}(t)$ at such times oscillates [7, 8] between positive and negative values, which means that the envelope of Ramsey fringes has points of zero amplitude where phase slips occur. In contrast, the average result is monotonically decaying.

Now we address the echo decay. In this case many thermal TLSs contribute and self-averaging, implied by Eq. (S7), is allowed. For the integration we need the function f_E in various limits. These are

$$\ln|f_E(t)| \approx \begin{cases} -\frac{v^2\Gamma t^3}{6} & \text{for } \; v\,t \ll 1\,,\, \Gamma\,t \ll 1\,, \\ -\Gamma t & \text{for } \; v\,t \gg 1\,,\, v \gg \Gamma\,, \\ -\frac{v^2 t}{\Gamma} & \text{for } \; \Gamma t \gg 1\,,\, \Gamma \gg v\,. \end{cases} \tag{S11}$$

Performing the integration we observe that the third domain of (S11), which corresponds mostly to the remote, weakly coupled TLS, does not contribute substantially. The integration over the two other domains gives

$$\Gamma_{\varphi,E} \approx \sqrt{J_T\,\Gamma_{1,T}^{\max}\,\xi^{-1}|\cos\theta_p|} \propto \sqrt{|\varepsilon_p|}\,. \tag{S12}$$

We note that in the considered limit, i.e., for nearly static thermal TLSs, the dephasing is substantially reduced by the echo technique. In fact the dephasing is even more reduced than observed in the experiments. Moreover, the functional dependence on $|\varepsilon_p|$ contradicts the experimental findings. Thus, other contributions to echo dephasing must be present.

To obtain the typical coupling J_T we estimate the typical distance, R_T, between the probed TLS and its nearest thermal TLS. For a bulk situation (3D) or a thin tunnel barrier (2D) of thickness d ($\approx 3\,$nm) we have

$$\begin{aligned} R_{T,3D}^3 \int_{u_{\min}}^1 du \int_0^T dE\,P(E,u) = 1 \quad (3D)\;\;, \\ R_{T,2D}^2\,d \int_{u_{\min}}^1 du \int_0^T dE\,P(E,u) = 1 \quad (2D)\;\;. \end{aligned} \tag{S13}$$

Substituting Eq. (S8) into Eqs. (S13), we readily obtain $R_{T,3D}^3 = R_{T,2D}^2\,d \approx (P_0\,\xi\,T)^{-1}$ with $\xi = \ln(1/u_{\min})$. Inserting this distance in the interaction coefficients Eq. (3), we find the typical coupling strength between the probed TLS and its nearest thermal TLS given in Eqs. (7).

D. Theory of pure dephasing due to non-equlibrium quasiparticles.

Quasiparticles in the superconducting electrodes constitute an additional source of decoherence. A quasiparticle scattering from a TLS in the amorphous layer of the junction disturbs the local electrical field at the TLS position and induces a slight asymmetry between the TLS potential wells. Hence, the coupling between quasiparticles and the TLS is dipole like, $H_{\rm qp} = g\,\sigma_z \sum_{k_1,k_2} c_{k_1}^\dagger c_{k_2}$, where the operators c_k stand for electron operators in the leads of the tunnel junction.

At the base temperature of the experiments, the number of quasiparticles and their effect should be frozen out. However, in previous works enhanced quasiparticle densities have been observed [13]. We therefore proceed assuming that a certain number of quasiparticles are present in a narrow energy window of width $\delta_{\rm qp}$ above the superconducting energy gap $\Delta_{\rm BCS}$. A typical experimentally observed value of the non-equilibrium quasiparticles density is $n_{qp} \sim 10^{-6} N_0 \Delta_{\rm BCS}$ [13], where n_{qp} is defined as

$$n_{qp} = 2N_0 \int_{\Delta_{\rm BCS}}^{\infty} dE \rho(E) f(E)\,. \tag{S14}$$

Here N_0 is the density of states at the Fermi level of the normal state, $\rho(E)$ is the reduced density of states in the superconductor, here assumed to be of the ideal BCS form $\rho(E) = E/\sqrt{E^2 - \Delta_{\rm BCS}^2}$, and $f(E)$ is the quasiparticle distribution function.

The resulting dephasing function for the Ramsey and spin-echo protocol has been analyzed in Ref. [14] with the results

$$x_{R/E} = \left(\frac{\epsilon_p}{E_p}\right)^2 2\pi N_0^2 g^2 \Delta_{\rm BCS} \Big[8 f_0 t + c_{R/E}\,(1-\cos\phi)\, f(\Delta_{\rm BCS})[\gamma_e - 1 + \log(4c_{R/E}^2 \delta t)]t\Big] \tag{S15}$$

The phase ϕ depends on the exact physical nature of quasiparticle scattering and will be discussed below. The difference between Ramsey and spin echo lies mostly in the value of the constant $c_{R/E}$, where $c_R = 1$ and $c_E = 1/2$. The decay function is highly sensitive to the distribution function at the gap $f(\Delta_{BCS})$ because of the divergence of the density of states. We also introduced the quantity $f_0 = \Delta_{\rm BCS}^{-1} \int_{\Delta_{\rm BCS}}^{\infty} f(E)\, dE = \delta f(\Delta_{\rm BCS})/\Delta_{\rm BCS}$, which does not contain the superconducting density of states due to a cancellation against the coherence factors. Using our assumption that the quasiparticles are distributed within an energy window $\delta_{\rm qp}$ above the gap, we can connect the number of quasiparticle n_{qp} to $f(\Delta_{\rm BCS})$ via $n_{qp} = 2N_0 \delta_{\rm qp} \sqrt{1 + 2\Delta_{\rm BCS}/\delta_{\rm qp}}\, f(\Delta_{\rm BCS})$.

The phase ϕ differs in two characteristic situations. The first is the case where quasiparticles from one electrode scatter at the TLS back into the same electrode. In this case we have $\phi = 0$. It is then immediately clear that, as for white noise, the Ramsey and echo signals are the same, $x_R/x_E = 1$. Our recent experiments with quasiparticle injection yield an estimate for the coupling constant g such that $16\pi N_0^2 g^2 \Delta_{\rm BCS} \approx 10^6 (\mu{\rm s})^{-1}$. With the quasiparticle number as given in Ref. [13], we find that $16\pi N_0^2 g^2 \Delta_{\rm BCS} f_0 \approx 0.1 - 1\,(\mu{\rm s})^{-1}$ which is of the same order of magnitude as the fitted values for the parameter A in Eq. (10).

Another situation is the case where quasiparticles tunnel through the junction and hence are sensitive to the superconducting phase difference $\phi \neq 0$ across the junction. In this case the decoherence is described by a decay law with an exponent $\propto t \ln(t)$. For long times, the logarithmic term dominates and the ratio between Ramsey and spin echo decay rates is given by $x_R(t \to \infty)/x_E(t \to \infty) = 2$.

Comparing the results obtained here with the experiments, we conclude that quasiparticles cannot explain the value of both the Ramsey and the echo decay rates. However, the value of the echo decay rate is of the right order of magnitude, and may be what is observed when the effects of thermal TLSs, which strongly influence the Ramsey signal, are suppressed.

[1] Steffen, M. *et al.*, State tomography of capacitively shunted phase qubits with high fidelity. *Phys. Rev. Lett.* **97**, 050502 (2005).
[2] Lisenfeld, J. *et al.*, Observation of directly interacting coherent two-level systems in an amorphous material. *Nat. Commun.* **6**, 6182 (2015).
[3] Lisenfeld, J. *et al.*, Rabi spectroscopy of a qubit-fluctuator system. *Phys. Rev. B* **81**, 100511(R) (2010).
[4] Neeley, M. *et al.*, Process tomography of quantum memory in a Josephson-phase qubit coupled to a two-level state. *Nature Physics* **4**, 523 (2008).
[5] Deppe, F. *et al.*, Phase coherent dynamics of a superconducting flux qubit with capacitive bias readout. *Phys. Rev. B* **76**, 214503 (2007).
[6] Paladino, E., Faoro, L., Falci, G. & Fazio, R. Decoherence and $1/f$ noise in josephson qubits. *Phys. Rev. Lett.* **88**, 228304 (2002).
[7] Galperin, Y., Altshuler, B. & Shantsev, D., Low-frequency noise as a source of dephasing of a qubit. In Lerner, I., Altshuler, B. & Gefen, Y. (eds.) *Fundamental Problems of Mesoscopic Physics*, vol. 154 of *NATO Science Series*, 141–165 (Springer Netherlands, 2004).
[8] Bergli, J., Galperin, Y. M. & Altshuler, B. L., Decoherence in qubits due to low-frequency noise. *New J. Phys.* **11**, 025002 (2009).
[9] Schriefl, J., Makhlin, Yu., Shnirman, A., & Sch"on, G. Decoherence from ensembles of two-level fluctuators. *New J. Phys.* **8**, 1 (2008).
[10] Hunklinger, S., Tunneling in amorphous solids. *Cryogenics* **28**, 224 – 229 (1988).

[11] Ithier, G. *et al.*, Decoherence in a superconducting quantum bit circuit. *Phys. Rev. B* **72**, 134519 (2005).
[12] Viola, L. & Lloyd, S., Dynamical suppression of decoherence in two-state quantum systems. *Phys. Rev. A* **58**, 2733–2744 (1998).
[13] Martinis, J. M., Ansmann, M. & Aumentado, J., Energy decay in superconducting josephson-junction qubits from nonequilibrium quasiparticle excitations. *Phys. Rev. Lett.* **103**, 097002 (2009).
[14] Zanker, S. & Marthaler, M., Qubit dephasing due to quasiparticle tunneling. *Phys. Rev. B* **91**, 174504 (2015).
